# Regional amyloid correlates of cognitive performance in ageing and mild cognitive impairment

**DOI:** 10.1093/braincomms/fcac016

**Published:** 2022-02-07

**Authors:** Daniel A. Stevens, Clifford I. Workman, Hiroto Kuwabara, Meryl A. Butters, Alena Savonenko, Najilla Nassery, Neda Gould, Michael Kraut, Jin Hui Joo, Jessica Kilgore, Vidya Kamath, Daniel P. Holt, Robert F. Dannals, Ayon Nandi, Chiadi U. Onyike, Gwenn S. Smith

**Affiliations:** 1 Division of Geriatric Psychiatry and Neuropsychiatry, Department of Psychiatry and Behavioral Sciences, School of Medicine, Johns Hopkins University, Baltimore, MD, USA; 2 Division of Nuclear Medicine and Molecular Imaging, Russell H. Morgan Department of Radiology and Radiological Sciences, School of Medicine, Johns Hopkins University, Baltimore, MD, USA; 3 Department of Psychiatry, University of Pittsburgh School of Medicine, Pittsburgh, PA, USA; 4 Department of Pathology (Neuropathology), School of Medicine, Johns Hopkins University, Baltimore, MD, USA; 5 Department of General Internal Medicine, School of Medicine, Johns Hopkins University, Baltimore, MD, USA; 6 Division of Neuroradiology, Russell H. Morgan Department of Radiology and Radiological Sciences, School of Medicine, Johns Hopkins University, Baltimore, MD, USA; 7 Department of Neurology, School of Medicine, Johns Hopkins University, Baltimore, MD, USA

**Keywords:** ageing, amyloid, cognition, mild cognitive impairment, positron-emission tomography

## Abstract

Beta-amyloid deposition is one of the earliest pathological markers associated with Alzheimer's disease. Mild cognitive impairment in the setting of beta-amyloid deposition is considered to represent a preclinical manifestation of Alzheimer's disease. *In vivo* imaging studies are unique in their potential to advance our understanding of the role of beta-amyloid deposition in cognitive deficits in Alzheimer's disease and in mild cognitive impairment. Previous work has shown an association between global cortical measures of beta-amyloid deposition (‘amyloid positivity’) in mild cognitive impairment with greater cognitive deficits and greater risk of progression to Alzheimer's disease. The focus of the present study was to examine the relationship between the regional distribution of beta-amyloid deposition and specific cognitive deficits in people with mild cognitive impairment and cognitively normal elderly individuals. Forty-seven participants with multi-domain, amnestic mild cognitive impairment (43% female, aged 57–82 years) and 37 healthy, cognitively normal comparison subjects (42% female, aged 55–82 years) underwent clinical and neuropsychological assessments and high-resolution positron emission tomography with the radiotracer ^11^C-labelled Pittsburgh compound B to measure beta-amyloid deposition. Brain–behaviour partial least-squares analysis was conducted to identify spatial patterns of beta-amyloid deposition that correlated with the performance on neuropsychological assessments. Partial least-squares analysis identified a single significant (*P* < 0.001) latent variable which accounted for 80% of the covariance between demographic and cognitive measures and beta-amyloid deposition. Performance in immediate verbal recall (*R* = −0.46 ± 0.07, *P* < 0.001), delayed verbal recall (*R* = −0.39 ± 0.09, *P* < 0.001), immediate visual-spatial recall (*R* = −0.39 ± 0.08, *P* < 0.001), delayed visual-spatial recall (*R* = −0.45 ± 0.08, *P* < 0.001) and semantic fluency (*R* = −0.33 ± 0.11, *P* = 0.002) but not phonemic fluency (*R* = −0.05 ± 0.12, *P* < 0.705) negatively covaried with beta-amyloid deposition in the identified regions. Partial least-squares analysis of the same cognitive measures with grey matter volumes showed similar associations in overlapping brain regions. These findings suggest that the regional distribution of beta-amyloid deposition and grey matter volumetric decreases is associated with deficits in executive function and memory in mild cognitive impairment. Longitudinal analysis of these relationships may advance our understanding of the role of beta-amyloid deposition in relation to grey matter volumetric decreases in cognitive decline.

## Teaser

Stevens *et al*. analysed cross-sectional ^11^C-labelled Pittsburgh compound B PET and neuropsychologic measures from people with multi-domain amnestic mild cognitive impairment and healthy comparison subjects using brain–behaviour partial least-squares analysis. They report on potential imaging correlates of Alzheimer's related impairment in verbal fluency, auditory-verbal and visual-spatial learning and memory.

## Graphical Abstract

**Graphical Abstract:**
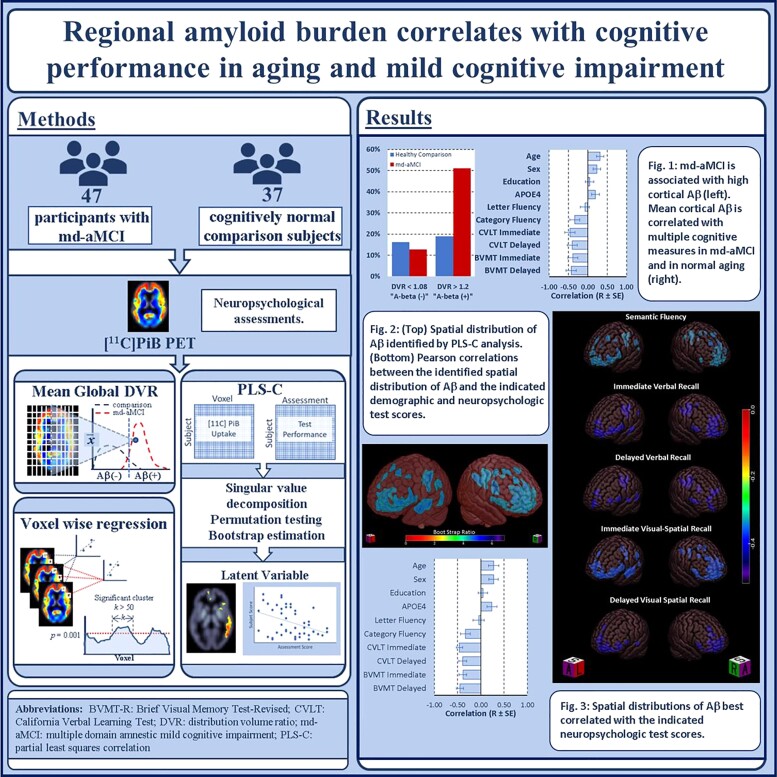

## Introduction

Alzheimer's disease is the most common cause of dementia, affecting an estimated 5.8 million people in the USA.^1^ Currently, there are no disease-modifying therapies for Alzheimer's disease, and without treatments to slow the underlying pathological processes, the prevalence of the disease is projected to reach 13 million by 2050.^1^ Extracellular deposition of beta-amyloid (Aβ) protein throughout multiple cortical and subcortical regions is one of the earliest pathological markers associated with Alzheimer's disease.^2^ Aβ accumulation begins decades before the appearance of clinical symptoms, triggering a pathological cascade leading to synaptic dysfunction, neuroinflammation and neuronal loss, all of which have been attributed to the irreversible cognitive and functional decline seen in Alzheimer's disease.^3–5^

Despite the role of Aβ in the pathophysiology of Alzheimer's disease, most pre- and post-mortem studies of mean cortical Aβ in Alzheimer's disease show limited correlation with the degree of cognitive impairment.^6,7^ In fact, up to 30% of cognitively healthy elderly individuals exhibit mean cortical Aβ in the range seen in people with Alzheimer's disease and do not have cognitive deficits relative to individuals without Aβ.^8,9^ Moreover, recent therapeutic attempts at targeting Aβ plaques have demonstrated limited success in curbing cognitive and functional decline.^10^ Potential reasons could include attempting these interventions too late in the disease course or challenges in targeting those most likely to benefit from these therapies. Identification of earlier or more specific Aβ-related correlates of cognitive decline may allow for more targeted intervention and better measurement of the true impact of anti-amyloid therapies on risk of further cognitive decline or progression to Alzheimer's disease.

Molecular imaging of Aβ *in vivo* using Aβ-selective radiotracers for PET offers unique advantages for detecting early disease, performing mechanistic studies and monitoring the effects of Aβ-targeted therapeutics. Longitudinal Aβ PET studies have found rates of Aβ accumulation to be a sensitive correlate of cognitive decline in Alzheimer's disease and its preclinical stages, and in ‘normal ageing’ populations.^11–15^ Amongst the available radiotracers, ^11^C-labelled Pittsburgh compound B ([^11^C]-PiB) is the best-characterized agent for imaging Aβ—exhibiting, high binding affinity and specificity, and strong correlation with post-mortem Aβ plaque distribution and other pertinent neuroimaging findings (e.g. cortical atrophy).^16–25^

Mild cognitive impairment (MCI) is defined as an objective impairment in at least one domain of cognitive function without significant impairment in activities of daily living. MCI is widely recognized as a preclinical manifestation of Alzheimer's disease, and an opportunity for targeted early intervention.^26^ In particular, deficits in episodic verbal and/or visual-spatial memory, amnestic MCI (aMCI) or single-domain amnestic MCI (sd-aMCI), have been associated with a higher likelihood of progression to Alzheimer's disease when compared with non-amnestic/dysexecutive MCI.^27,28^ The risk of progression to Alzheimer's disease is even greater in the sub-group of individuals who exhibit cognitive deficits in multiple domains (md-aMCI) compared with those with only deficits in episodic memory.^27,29–32^ These MCI subtypes may represent a continuum in the progression to Alzheimer's disease and identification of the earliest molecular events predicting enrolment in this continuum will be key to validating primary preventive interventions for both MCI and Alzheimer's disease.

The relationship between the magnitude and localization of Aβ deposition to cognitive impairment in MCI is not well understood. Molecular imaging studies of Aβ in MCI suggest that, on average, individuals with MCI exhibit mean cortical levels of Aβ between those of healthy controls and Alzheimer's disease patients.^8,9^ However, there appears to be a bimodal distribution in the magnitude of mean cortical Aβ within individuals with MCI—some exhibiting high Aβ, and others showing very little Aβ, independent of their degree of cognitive impairment.^8,9,33^ Many studies use cut-off values of mean cortical or global Aβ to designate an individual as ‘amyloid positive’ or ‘amyloid negative’. The designation of ‘amyloid positivity’ has been associated with the risk of progression to Alzheimer's disease, as well as the nature of cognitive deficits in MCI. Whilst people with aMCI are 2–3 times more likely to exhibit high Aβ, a limited number of studies have found md-aMCI to be associated with rates of Aβ positivity ranging from 77 to 83%, providing further evidence of a preclinical Alzheimer's disease continuum.^27,28,30,31^

Contrary to observations in Alzheimer's disease, many studies of those with MCI have found that mean cortical Aβ is associated with cognitive scores, particularly measures of episodic memory.^6,8,34–36^ Some but not all studies have extended these findings to cognitively normal older adults.^23,37–40^ This relationship appears to be modulated by Apolipoprotein E4 (*APOE4*) genotype and older age, amongst other factors.^38^ However, correlation between the spatial distribution of Aβ and cognitive performance has not been a major focus of investigation. Understanding the covariance between the spatial distribution of Aβ and cognitive performance in normal ageing and early MCI could help guide future longitudinal mechanistic studies and may yield imaging correlates for early Alzheimer's disease-related cognitive decline that is more sensitive than mean cortical Aβ or CSF measures of Aβ.

To study the spatial distribution of Aβ and its correlation to cognition, robust data-driven approaches are needed. Commonly used univariate and region of interest approaches may overlook more complex patterns in the data. Whole-brain voxel-wise analyses are limited by the sheer number of comparisons and the consequent risk for false-positive findings. The application of data-driven multivariate approaches [e.g. partial least-squares (PLS)] addresses the issue of multiple comparisons and allows for the elucidation of complex patterns of covariance contributing to variation in the nature and severity of cognitive impairment.

The purpose of this study was to evaluate the regional distribution of Aβ and its correlations with neuropsychological measures in a group of individuals with md-aMCI and a demographically matched, healthy comparison group. The hypotheses tested were that (i) md-aMCI patients would show greater Aβ in frontal, temporal and parietal association cortices relative to the comparison group and (ii) greater Aβ would correlate with poorer neuropsychological performance in memory and executive function.

## Materials and methods

### Participant screening and selection

Participants were recruited from advertisements in the community or from the Johns Hopkins University Alzheimer's Disease Research Center (2P50AG005146). Participants underwent a physical and neurological examination, laboratory testing and toxicology screening, psychiatric screening (Structured Clinical Interview for DSM-IV) and global cognitive and functional evaluation [Clinical Dementia Rating (CDR)] Scale.^41,42^ A comprehensive multi-domain neuropsychological test battery was administered to all prospective participants, which included the Wechsler Test of Adult Reading, the Delis–Kaplan Executive Function System (DKEFS), the California Verbal Learning Test (CVLT), the Wechsler Memory Scale-Third Edition, the Brief Visual Memory Test-Revised (BVMT-R) and the Symbol Digit Modalities Test.^43–48^ Enrolment into the md-aMCI arm required that subjects score 0.5 on the CDR and also, at least 1 SD below the mean on the CVLT or BVMT-R and on at least one executive function test. Subjects in the comparison group had to have a CDR score of 0.

Chronic health conditions such as hypertension and diabetes had to be well-controlled in all participants. Subjects were excluded from the study if they had a current neurological disorder or a DSM-V diagnosis of a psychiatric disorder (except neurocognitive disorder in the md-aMCI participants), contraindications for MRI (e.g. pacemakers, cerebral arterial aneurysm clips or metal implanted in the body) or if they were prescribed a psychotropic medication and/or had a positive toxicology screen for psychotropic drugs or medications with central nervous system effects (e.g. antihistamines, cold medications) in the 2 weeks prior to enrolment. The study protocol and consent forms were approved by the Johns Hopkins Institutional Review Board and all participants gave written informed consent.

### Genotyping

ApoE genotyping was performed using polymerase chain reaction amplification of genomic DNA digestion with Hhal restriction enzyme and gel electrophoresis in the laboratory of Dimitri Avramopoulos, MD, PhD, Institute for Genetic Medicine, Johns Hopkins University School of Medicine, Baltimore, MD, USA.^49,50^

### MR imaging procedures and processing

MRI scans of the brain were acquired before the PET as previously described.^51^ A Phillips 3.0 T Achieva MRI instrument was used with an eight-channel head coil (Philips Medical Systems, Best, The Netherlands) at the F. M. Kirby Research Center for Functional Brain Imaging of the Kennedy Krieger Institute. The magnetization-prepared rapid acquisition with gradient-echo (MPRAGE) pulse sequence (time to echo = 4, repetition time = 8.9, flip angle = 8°, number of signals averaged = 1, 0.7 mm isotropic voxel size) was used for volumes of interest (VOI) delineation and PET image processing. MPRAGE files were segmented using Freesurfer (v6.0), generating grey matter probability maps and volumes of interest. Grey matter probabilistic maps were registered to standard Montreal Neurologic Institute (MNI) space. In the presence of atrophy, grey matter voxels in the native MRI space are ‘expanded’ by spatial normalization, thus, the more atrophy, the lower GM intensity in individual voxels in the standard space. These normalized grey matter probabilistic maps were smoothed with a 10 mm Gaussian kernel prior to PLS analysis for comparison with PET image resolution.

### PET imaging procedures, preprocessing and tracer kinetic modelling

PET scans were performed at the PET Center of the Russell H. Morgan Department of Radiology, Johns Hopkins University School of Medicine. The scanner was a second-generation High-Resolution Research Tomograph scanner (HRRT, Siemens Healthcare, Knoxville, TN, USA), a cerium-doped lutetium oxyorthosilicate (Lu25i05[Ce] or LSO) detector-based dedicated brain PET scanner. Each subject was fitted with a thermoplastic mask moulded to his/her face to reduce head motion during the PET study. Attenuation maps were generated from a 6-min transmission scan performed with a [^137^Cs] point source prior to the emission scans.

N-methyl-[11C]2-(4′-methylaminophenyl)-6-hydroxybenzothiazole ([^11^C]-PiB) was synthesized as previously described.^18^ Dynamic scanning with the radiotracer began immediately upon a 15 mCi ± 10% radiotracer injection and lasted 90 minutes. Data were acquired in list mode. The images were reconstructed using the iterative ordered subset-expectation maximization (OS-EM) algorithm (with 6 iterations and 16 subsets), with correction for radioactive decay, dead time, attenuation, scatter and randoms, and re-binned into 30 frames (four 15 seconds, four 30 seconds, three 1 minute, two 2 minute, five 4 minute and twelve 5 minute frames).^52^ The reconstructed image space consisted of 256 (left-to-right) by 256 (nasion-to-inion) by 207 (neck-to-cranium) cubic voxels, each 1.22 mm in dimension. The final spatial resolution was <2.5 mm full width at half-maximum in three directions.^53^

Preprocessing of parametric [^11^C]-PiB distribution volume ratio (DVR) images was performed with Statistical Parametric Mapping software (SPM12; Institute of Neurology, London) as described previously.^54^ Regional DVR values of [^11^C]-PiB were calculated using a multilinear reference tissue method with two parameters with cerebellar grey matter (excluding vermis) as reference region.^55–57^ Mean cortical Aβ values were calculated based on FreeSurfer (v6.0) determined VOI.^58^

### Statistical analyses

One-way ANOVA was used to assess the difference in demographic, clinical and neuropsychological variables between groups. χ^2^ tests were used to assess for between-group differences in sex and ApoE genotype distribution. Two mean cortical Aβ cut-off values that have been validated with post-mortem data are reported for comparison with prior studies.^54,59^ Specifically, we quantified subjects with: (i) mean cortical DVR ≤ 1.08, a value associated with a 90% likelihood of being a low Aβ accumulator and (ii) mean cortical DVR ≥ 1.20 associated with a 90% likelihood of being a high Aβ accumulator.^59^ ANOVA was performed to assess whether Aβ status (positive/negative) was associated with differences in neuropsychological performance. We evaluated subjects against these cut-offs for comparison with previous literature and to assess the correlation of cognition with global mean Aβ in this sample. We did not exclude any subject from the remainder of the analyses. Not excluding ‘amyloid negative’ participants in our view would make our findings more widely applicable. We used *t*-tests to compare mean cortical Aβ between groups. We performed simple regression comparing continuous mean cortical Aβ DVR with DKEFS Category and Letter Fluency scores and the scores of all immediate and delayed recall trials from the CVLT and BVMT-R.

### Voxel-wise group comparison

Voxel-wise statistical analyses of parametric [^11^C]-PiB DVR images and Freesurfer generated, spatially normalized, smoothed, grey matter probability maps were performed with SPM12 (Institute of Neurology, London) as described previously.^54^ An absolute threshold of 0.2 was applied to the grey matter maps. Two sample *t*-tests tested for differences in Aβ and grey matter volumes between md-aMCIs and the comparison group. The significance criteria for reporting the SPM results were set at a cluster-level, family-wise error corrected threshold of *P* ≤ 0.001, and a peak voxel corrected threshold of *P* ≤ 0.0001: height threshold *P* = 0.0001 and extent threshold (*k*) = 50 voxels.

### PLS analysis

Brain–behaviour partial least-squares correlation (PLS-C) analysis was conducted (software available from http://pls.rotman-baycrest.on.ca/source/e) using smoothed PET images and grey matter probabilistic maps normalized to MNI space for all participants combined. Behavioural variables included in the analysis were age, sex, years of education, number of *APOE4* alleles, DKEFS Category and Letter Fluency scores and the total scores of immediate and delayed free recall from the CVLT and BVMT-R. The mathematical details and application of PLS to neuroimaging data have been described previously.^60,61^ Briefly, singular value decomposition of imaging and behavioural data matrices facilitates identification of latent variables, each consisting of a topographic profile and behavioural profile which explain maximal covariance between these matrices. The number of latent variables generated is determined by the number of experimental groups and behavioural outcomes of interest entered into the analysis. Subject scores—the degree to which an individual exhibits the topographic profile given by a latent variable—can thus be correlated with covariates, in our case neuropsychological performance on selected tests resulting in Pearson coefficients of correlation (*R*).

Statistical validation was accomplished using two complementary resampling techniques. The behavioural salience of a particular latent variable was determined by running 100 permutations tests of the data without replacement. Latent variables were deemed significant if <5% of the permutations arrived at a greater singular value than the original result. Secondly, the reliability of each voxel's contribution to the latent variable was assessed using 1000 bootstrap iterations to arrive at estimates of standard errors for the voxel saliences. The use of bootstrap estimation of standard errors eliminates the need to correct for multiple comparisons because the voxel saliences are calculated in a single mathematical step, on the whole brain at once. Thresholds (and significance criteria) are established based on the variability observed in the bootstrapping. Correlation maps were generated for each neuropsychological measure. Voxels exhibiting significant correlation between intensity and individual cognitive measures [i.e. the 95% confidence interval of R, estimated by bootstrapping, does not cross zero] were extracted and mapped from MNI space to Talairach space. Clusters exceeding 50 contiguous correlated voxels were identified and the Brodmann area and mean correlation coefficients for the peak voxel in each cluster were reported.^62^

### Data availability

The data that support the findings of this study are available from the corresponding author, upon reasonable request.

## Results

### Between-group comparison of demographic data and neuropsychologic scores

Forty-seven participants with md-aMCI and 36 healthy comparison subjects completed the study procedures. Two participants in the md-aMCI group, and one participant in the comparison group was left handed. The groups did not significantly differ in age [*t*(82) = −1.44, *P* = 0.155], sex distribution [*X*(36,47) = 0.145, *P* = 0.703] or in level of education [*t*(82) = 0.450, *P* = 0.654]. There was no significant difference in the distribution of ApoE genotypes [*X* = (36,47) = 0.008, *P* < 0.100]. Subjects with md-aMCI scored significantly higher on the CDR sum of boxes [*F*(1,83) = 42.19, *P* < 0.001] and lower on the Mini-Mental State Exam [*F*(1,83) = 12.53, *P* < 0.001]. Representative outcome measures for the neuropsychological assessments are listed in Supplementary Table 1, including measures selected *a priori* for correlation with Aβ. Participants with md-aMCI scored significantly lower than comparison subjects on measures of verbal immediate [CVLT; *F*(1,83) = 19.36, *P* < 0.001] and delayed recall [CVLT; *F*(1,83) = 22.86, *P* < 0.001], visual-spatial immediate [BVMT-R; *F*(1,83) = 5.14, *P* = 0.026] and delayed recall [*F*(1,83) = 8.15, *P* = 0.005], Category Fluency [DKEFS: *F*(1,83) = 12.40, *P* = 0.001] and Letter Fluency [DKEFS: *F*(1,83) = 7.97, *P* = 0.006] (Table 1).

**Table 1:** Demographic and clinical characteristics of subjects by group

	Mild cognitive impairment group (*n* = 47)	Healthy comparison group (*n* = 37)
Age	69 ± 7	67 ± 7
Sex (F/M)	20/27	15/21
Education (in years)	15 ± 3	16 ± 3
Clinical Dementia Rating sum of boxes (CDR-SB)	1.20 ± 0.83***	0
Mini-Mental State Examination (MMSE)	28 ± 2***	29 ± 1
Apolipoprotein E genotyping		
2,3	6	3
3,3	24	23
3,4	9	7
4,4	4	3
Delis–Kaplan Executive Function Test, Letter Fluency	37.47 ± 10.94**	44.68 ± 12.51
Delis–Kaplan Executive Function Test, Category Fluency	33.83 ± 9.65**	40.68 ± 8.24
California Verbal Learning Test, total recall trials 1–5	44.17 ± 12.20***	55.22 ± 10.71
California Verbal Learning Test, long delay free recall	8.17 ± 3.92***	11.89 ± 2.98
Brief Visual Memory Test-revised, total recall trials 1–3	15.30 ± 7.84*	18.92 ± 6.09
Brief Visual Memory Test-revised, delayed recall	5.81 ± 3.17**	7.68 ± 2.58

* *P* < 0.05.

** *P* < 0.01.

*** *P* < 0.001.

### Between-group comparison of Aβ and grey matter volumes

The md-aMCI group had a mean cortical DVR of 1.41 ± 0.06 compared with 1.17 ± 0.03 for the comparison group [*t*(83) = −1.44, *P* < 0.001] (Supplementary Fig. 1). Six of 47 (12.8%) md-aMCI subjects and 6 of 37 (16.2%) comparison subjects had a mean cortical DVR ≤ 1.08. Twenty-four of 47 (51.1%) md-aMCI subjects and seven of 37 (18.9%) comparison subjects exceeded a cortical DVR ≥ 1.2 (Supplementary Fig. 1). Supplementary Table 2 lists the brain regions in which md-aMCIs subjects had significantly greater Aβ relative to the comparison group. There were no regions in which greater Aβ was found in the comparison group relative to the md-aMCI group. There were no statistically significant differences in regional grey matter volumes when comparing md-aMCI subjects to the comparison group.

### Brain–behaviour PLS analysis and voxel-wise regression

#### Amyloid PET

**Figure 1:**
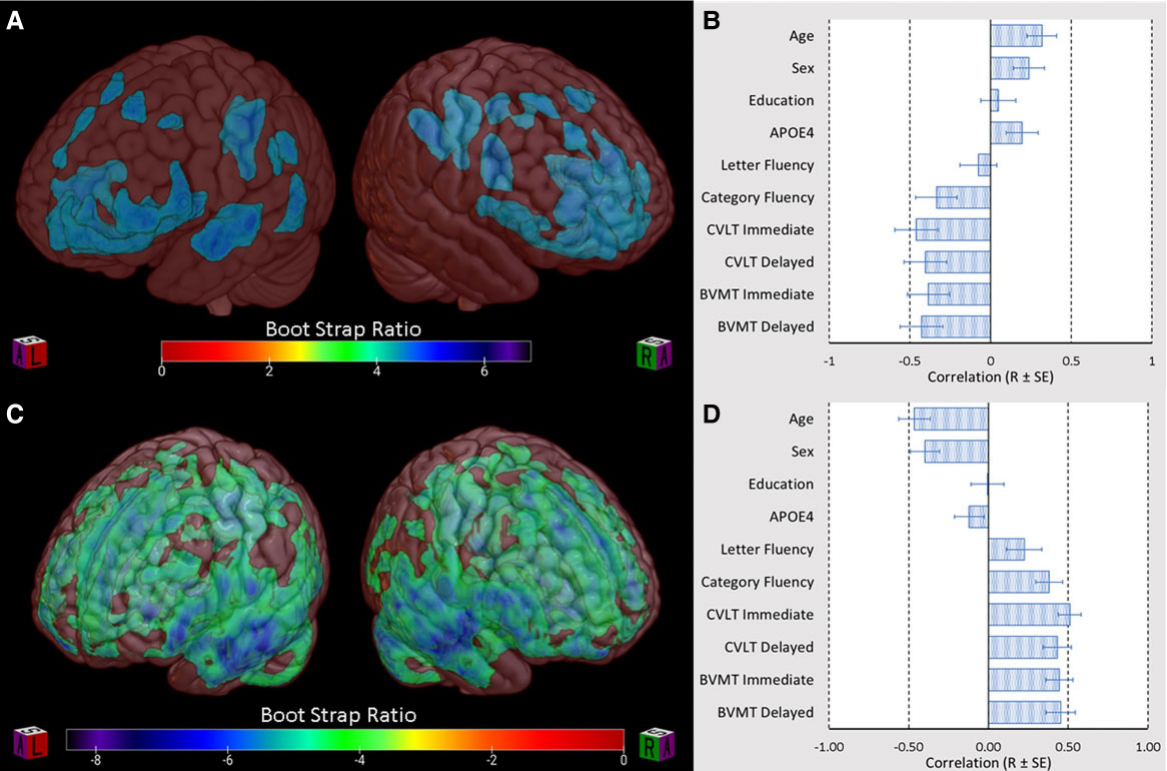
**Latent variables identified by PLS analysis**. (**A** and **C**) BSR maps depicting voxels which make a reliable contribution to latent variable one, thus representing the spatial distribution of (**A**) Aβ and (**C**) grey matter atrophy most strongly correlated with demographic variables and cognitive performance. (**B** and **D**) Bar graphs representing bootstrap estimated Pearson correlations with standard error (*R* ± *SE*) of the latent variable identified in (**B**) Aβ PLS and (**D**) grey matter volume PLS with select demographic variables and cognitive performance. (**B**) The first latent variable of the Aβ analysis was correlated with age (*R* = 0.27 ± 0.11, *P* = 0.014), male sex (*R* = 0.27 ± 0.10, *P* = 0.006), number of *APOE4* alleles (*R* = 0.24 ± 0.11, *P* = 0.028), immediate verbal recall (*R* = −0.46 ± 0.07, *P* < 0.001), delayed verbal recall (*R* = −0.39 ± 0.09, *P* < 0.001), immediate visual-spatial recall (*R* = −0.39 ± 0.08, *P* < 0.001), delayed visual-spatial recall (*R* = −0.45 ± 0.08, *P* < 0.001) and Category Fluency (*R* = −0.33 ± 0.11, *P* = 0.002) but not Letter Fluency (*R* = −0.05 ± 0.12, *P* = 0.705) years of education (*R* = 0.04 ± 0.10, *P* = 0.676). (**D**) The first latent variable of the grey matter volumetric analysis correlated with age (*R* = −0.46 ± 0.10, *P* < 0.001) and male sex (*R* = −0.40 ± 0.09, *P* < 0.001), immediate verbal recall (*R* = 0.51 ± 0.07, *P* < 0.001), delayed verbal recall (*R* = 0.43 ± 0.09, *P* < 0.001), immediate visual-spatial recall (*R* = 0.44 ± 0.09, *P* < 0.001), delayed visual-spatial recall (*R* = 0.45 ± 0.09, *P* < 0.001), Category Fluency (*R* = 0.38 ± 0.08, *P* < 0.001) and Letter Fluency (*R* = 0.22 ± 0.11, *P* = 0.044) but not years of education (*R* = 0.00 ± 0.10, *P* = 0.968) or number of *APOE4* alleles (*R* = −0.12 ± 0.09, *P* = 0.201). BVMT-R, Brief Visual Memory Test-Revised; CVLT, California Verbal Learning Test.

**Figure 2:**
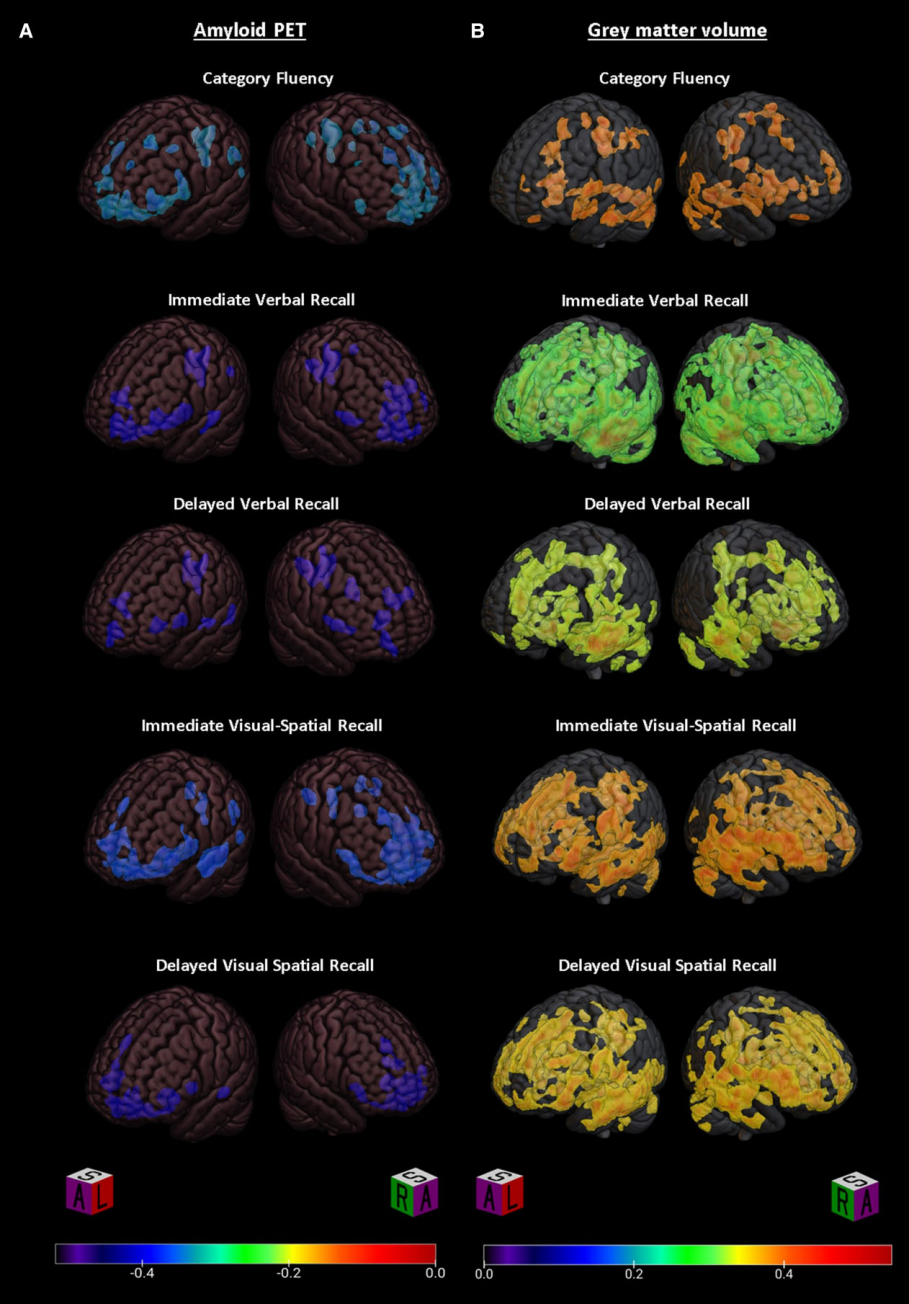
**Datamat correlation maps for individual cognitive measures**. Datamat correlation maps depicting voxels exhibiting significant correlation (*R*) of the indicated cognitive scores with (**A**) Aβ-PET or (**B**) grey matter probability.

PLS analysis of [^11^C]PiB-PET images from the combined subject sample identified a single latent variable which accounted for 83% of the cross-block variability between behavioural measures and Aβ. The topographic distribution of Aβ represented by this latent variable is shown in Fig. 1A. Subject scores, the degree to which an individual subject exhibits the identified topographic distribution, inversely correlated with performance in immediate verbal recall (*R* = −0.46 ± 0.07, *P* < 0.001), delayed verbal recall (*R* = −0.39 ± 0.09, *P* < 0.001), immediate visual-spatial recall (*R* = −0.39 ± 0.08, *P* < 0.001), delayed visual-spatial recall (*R* = −0.45 ± 0.08, *P* < 0.001) and Category Fluency (*R* = −0.33 ± 0.11, *P* = 0.002) but not Letter Fluency (*R* = −0.05 ± 0.12, *P* = 0.705) (Fig. 1B). Age (*R* = 0.27 ± 0.11, *P* = 0.014), male sex (*R* = 0.27 ± 0.10, *P* = 0.006) and number of *APOE4* alleles (*R* = 0.24 ± 0.11, *P* = 0.028) but not years of education (*R* = 0.04 ± 0.10, *P* = 0.676) were positively correlated with Aβ in the regions identified by latent variable one (Fig. 1B). Clusters exceeding 50 contiguous significant (i.e. bootstrap generated confidence intervals that did not cross zero) voxels were extracted and the peak voxels for these clusters, along with BSR are listed in Table 2. Datamat correlation maps for each neuropsychological outcome that was significantly correlated with latent variable one are shown in Fig. 2A. Table 3 lists the Pearson correlation score (*R*) for the peak voxels of clusters exceeding 50 contiguous significant voxels. Voxel-wise multivariate regression (SPM12) identified numerous clusters in the frontal, temporal and parietal cortical regions that correlated with semantic fluency performance, CVLT and BVMT-R scores in the combined md-aMCI and comparison groups—revealing substantial overlap with regions implicated by PLS (data not shown).

**Table 2:** Peak voxels for brain regions in which Aβ deposition is correlated with cognitive performance

Region	Left hemisphere			Right hemisphere		
	MNI coordinates *X Y Z* (mm)	Talairach coordinates *X Y Z* (mm)	BSR	MNI coordinates *X Y Z* (mm)	Talairach coordinates *X Y Z* (mm)	BSR
Frontal cortex						
Frontal eye fields (BA 8)				14 36 46	14 37 42	5.26
Pre/Supp. motor (BA 6)	−14 20 56	−13 23 51	5.74	14 16 60	14 20 54	5.32
Pre/Supp. motor (BA 6)	−30 8 52	−30 10 48	5.60			
Vent. Ant. cingulate (BA 24)				4 −18 38	4 −16 36	4.92
Insula (BA 13)				34 12 2	32 9 5	5.70
Temporal cortex						
Medial temporal gyrus (BA 21)	−54 −8 −18	−51 −11 −12	6.38			
Parietal cortex						
Angular gyrus (BA 39)	−44 −60 32	−44 −58 31	5.51			
Precuneus (BA 7)				14 −64 32	14 −61 30	6.08
Occipital cortex						
Visual association cortex (BA 19)	−48 −64 0	−47 64 5	6.50			
Sub-cortical regions						
Putamen	16 10 −6	−16 7 −2	6.88			

**Table 3:** Correlation coefficients for peak voxels in cortical regions in which Aβ deposition is correlated with individual cognitive measures

Region	Semantic fluency		CVLT immediate recall		CVLT delayed recall		BVMT-R immediate recall		BVMT-R delayed recall	
	Left	Right	Left	Right	Left	Right	Left	Right	Left	Right
Frontal cortex										
Pre/Supp. motor (BA 6)	−0.43	—	—	—	—	—	—		−0.44	—
Frontal eye fields (BA 8)	−0.36	—	—	—	—	—	—	−0.47	—	—
Dorsal lateral PFC (BA 9)	—	—	—	—	—	—	—	—	—	−0.46
Anterior PFC (BA 10)	−0.33	—	—	—	—	−0.40	—	—	—	—
Orbitofrontal (BA 11)	—	—	−0.45	—	—	—	—	—	−0.46	—
Insula (BA 13)	−0.32	—	—	−0.46	—	−0.41	—	−0.41	−0.44	—
Broca's operculum (BA 44)	—	—	—	—	−0.40	—	—	—	—	—
Dorsal lateral PFC (BA 46)	−0.38	−0.44	−0.44	—	—	—	—	—	—	—
Pars orbitalis (BA 47)	—	−0.34	—	—	—	—	—	—	—	—
Temporal cortex										
Medial temporal gyrus (BA 21)	—	—	−0.46	—	—	—	−0.45	—	−0.44	—
Sup. temporal gyrus (BA 22)	—	—	—	—	−0.40	—	—	—	—	—
Parietal cortex										
Dorsal post. cingulate (BA 31)	−0.33	−0.34	−0.46	—	—	−0.39	−0.39	—	—	—
Fusiform (BA 37)	—	—	—	—	−0.40	—	—	—	—	—
Angular gyrus (BA 39)	−0.36	—	−0.45	—	—	—	−0.37	—	—	—
Occipital cortex										
Visual association (BA 19)	—	—	—	—	—	—	−0.42	−0.41	—	—
Subcortical										
Putamen	—	—	—	—	−0.43	—	—	—	—	—

#### Grey matter volume

PLS analysis of grey matter probability images from the combined subject sample identified a single latent variable which accounted for 88% of the cross-block variability between behavioural measures and grey matter distribution. The topographic variability in grey matter probability represented by this latent variable is shown in Fig. 1C. Subject scores positively correlated with performance in immediate verbal recall (*R* = 0.51 ± 0.07, *P* < 0.001), delayed verbal recall (*R* = 0.43 ± 0.09, *P* < 0.001), immediate visual-spatial recall (*R* = 0.44 ± 0.09, *P* < 0.001), delayed visual-spatial recall (*R* = 0.45 ± 0.09, *P* < 0.001), semantic fluency (*R* = 0.38 ± 0.08, *P* < 0.001) and phonemic fluency (*R* = 0.22 ± 0.11, *P* = 0.044) (Fig. 1D). Age (*R* = −0.46 ± 0.10, *P* < 0.001) and male sex (*R* = −0.40 ± 0.09, *P* < 0.001), but not or years of education (*R* = 0.00 ± 0.10, *P* = 0.968) or number of *APOE4* alleles (*R* = −0.12 ± 0.09, *P* = 0.201) negatively correlated with grey matter probability in the regions identified by latent variable one (Fig. 1D). Clusters exceeding 50 contiguous significant (i.e. bootstrap generated confidence intervals that did not cross zero) voxels were extracted and the peak voxels for these clusters, along with BSR are listed in Table 4. Datamat correlation maps for each neuropsychological outcome that was significantly correlated with latent variable one are shown in Fig. 2B. Correlation map clusters exceeding 50 contiguous significant (based on established BSR cut-offs) voxels were extracted and the Pearson correlations (*R*) of the peak voxels are listed by cognitive measure in Table 5.

**Table 4:** Peak voxels for brain regions in which grey matter volume is correlated with cognitive performance

Region	Left hemisphere			Right hemisphere		
	MNI coordinates *X Y Z* (mm)	Talairach coordinates *X Y Z* (mm)	BSR	MNI coordinates *X Y Z* (mm)	Talairach coordinates *X Y Z* (mm)	BSR
Frontal cortex						
Frontal eye fields (BA 8)	−4 24 46	−3 35 −42	−6.87			
Frontal eye fields (BA 8)	−30 20 54	−29 22 49	−5.98			
Dorsal lateral PFC (BA 9)	−44 22 28	−43 21 27	−5.89			
Orbitofrontal (BA 11)				6 36 −28	5 30 −26	−5.94
Temporal cortex						
Inf. temporal gyrus (BA 20)	−62 −34 −26	−58 −36 −19	−8.64			
Medial temporal gyrus (BA21)				66 −8 −14	62 −11 −7	−7.57
Hippocampus				18 −8 −20	17 −10 −13	−7.22
Parietal cortex						
Precuneus (BA 7)				34 −74 46	35 −70 42	−5.65
Dorsal PCC (BA 31)	−2 −50 38	−1 −47 36	−7.93			
Angular gyrus (BA 39)	−42 −54 50	−42 −51 46	−6.08	52 −62 36	53 −59 35	−5.44
Sub-cortical regions						
Caudate				8 10 4	7 7 6	−5.40

**Table 5:** Correlation coefficients for peak voxels in cortical regions in which grey matter volumes are correlated with individual cognitive measures

Region	Semantic fluency		CVLT immediate recall		CVLT delayed recall		BVMT-R immediate recall		BVMT-R delayed recall	
	Left	Right	Left	Right	Left	Right	Left	Right	Left	Right
Frontal cortex										
Frontal eye fields (BA 8)	—	—	—	0.46	0.42	0.46	0.37	0.37	0.44	0.41
Dorsal lateral PFC (BA 9)	—	—	—	—	0.36	—	0.43	—	—	—
Anterior PFC (BA 10)	0.34	—	—	—	0.41	—	0.36	—	—	—
Orbitofrontal (BA 11)	—	0.45	—	—	—	0.43	—	—	0.40	—
Broca's triangle (BA 45)	0.36	—	—	—	0.35	—	—	—	—	—
Pars orbitalis (BA 47)	—	—	—	—	0.42	—	—	—	—	—
Temporal cortex										
Medial temporal gyrus (BA 21)	0.43	0.25	—	—	—	—	—	—	—	—
Sup. temporal gyrus (BA 22)	—	—	—	—	—	0.38	—	—	—	—
Parahippocampus (BA 36)	—	—	—	—	—	—	—	—	0.45	—
Hippocampus	0.32	0.39	—	—	—	0.39	—	0.47	—	0.46
Parietal cortex										
Primary sensory (BA 1)	—	—	0.23	0.40	—	—	—	—	—	—
Precuneus (BA 7)	—	—	0.28	—	—	—	0.40	—	—	—
Dorsal post. cingulate (BA 31)	0.44	—	—	—	—	—	0.47	—	0.47	—
Fusiform (BA 37)	0.41	—	—	—	—	—	—	—	—	—
Angular gyrus (BA 39)	0.33	—	—	0.41	—	—	—	—	0.41	0.41
Supramarginal gyrus (BA 40)	—	—	0.36	—	—	—	—	—	—	0.42
Occipital cortex										
Secondary visual (BA 18)	—	—	—	—	—	—	—	0.32	—	—
Visual association (BA 19)	0.44	0.15	—	—	0.38	—	—	—	—	—
Subcortical										
Caudate	—	—	—	0.43	0.36	0.39	—	—	—	—
Thalamus	—	—	—	—	—	0.42	—	—	—	—

## Discussion

The focus of the study was to apply a data-driven multivariate approach, brain–behaviour PLS, to understand the associations between regional Aβ and performance in executive function and memory tasks in both md-aMCI and a comparison group of healthy older adults. We hypothesized that (i) md-aMCI patients would show greater Aβ in frontal, temporal and parietal association cortices relative to the comparison group and (ii) greater Aβ would correlate with poorer neuropsychological performance in memory and executive function. We found that the md-aMCI cohort showed deficits on measures of global cognition and multiple neuropsychological measures, including immediate and delayed memory, executive function and processing speed relative to the comparison group (Supplementary Table 1). The md-aMCI group also showed greater Aβ in the frontal, temporal and parietal association cortices, and in the putamen (Supplementary Table 2). For all subjects, mean cortical Aβ was negatively correlated with performance in Category Fluency, verbal immediate and delayed recall and visual-spatial immediate and delayed recall. Brain–behaviour PLS analysis of all participants combined identified multiple regions in which the degree of Aβ was negatively correlated with performance in select verbal and visual-spatial memory tests as well as Category Fluency (Table 3). Further, using the same analysis method, grey matter volumes in similar brain regions were positively correlated with performance on the same cognitive tests.

The application of data-driven approaches to examine associations between regional Aβ deposition and cognition in Alzheimer's disease or in its preclinical stages has been limited. In Alzheimer's disease, studies using mean cortical Aβ or Aβ in select regions of interest have found limited associations between the degree of Aβ and cognitive function.^6,7^ One independent component analysis that evaluated the utility of regional uptake of [^11^C]-PiB in distinguishing between Alzheimer's disease patients with predominately amnestic/dysexecutive, and those with language or visual-spatial deficits, found no regional Aβ patterns that discriminated between these sub-groups.^63^

Mean cortical Aβ has been associated with cognitive performance in MCI, whilst some but not all studies have identified correlations amongst normal ageing populations.^37,38^ However, studies of regional Aβ in MCI or healthy elderly individuals are limited. We observed cross-sectional correlations of Aβ with memory and Category Fluency (Table 2). A previous study conducted by Koivunen *et al*.^64^ used an automated region of interest approach to examine the correlation between cognitive performance and regional Aβ in subjects with MCI who did or did not progress to Alzheimer's disease over the course of 2 years. In contrast to our findings, they did not observe cross-sectional correlations between regional Aβ and any cognitive measure. There are many methodological differences that could contribute to these differences in results. For instance, our inclusion of both healthy comparison subjects and subjects with md-aMCI provided variability in both Aβ and cognitive performance, the difference in analytic methods (automated ROI versus PLS) and the greater sample size of our study.

Notably, Koivunen *et al*.^64^ did observe relationships between baseline regional Aβ and subsequent cognitive decline. Specifically, Aβ in the lateral frontal regions and in the caudate and putamen was associated with a decline in verbal memory measures over the 2-year interval. The decline in clock drawing performance was associated with higher baseline Aβ in the medial frontal regions and in the anterior cingulate cortex, and higher temporal cortex Aβ was associated with decline in Stroop interference scores. This study did not find an association between Aβ and decline in semantic fluency and did not include tests of visual-spatial memory. There is a limited overlap of these observations with our cross-sectional results; however, ongoing longitudinal studies in this cohort may provide greater insight on how these findings are related.

In this cohort, we observe that all of the assessed cognitive measures contribute to variability along a single latent variable in both the Aβ and the grey matter volume analyses. This may be indicative of overlap between these cognitive measures in terms of mental processes and brain networks recruited. For example, the study of visual-spatial memory in MCI and Alzheimer's disease may be complicated by deficits in verbal memory, fluid ability and processing speed which all influence task performance.^65,66^ It may also partly reflect the convergence of Aβ into a more consistent Alzheimer's distribution as total Aβ increases.

Analysis of Aβ correlation maps for the individual cognitive measures suggests some specific regional correlations with each measure (Table 3). The cross-sectional nature of this analysis means that we could only speculate on the underlying processes driving these differential correlations. One might speculate, for instance, that in some brain regions, Aβ accumulation may contribute to progressive local network dysfunction and impaired cognition in a specific task. Category (semantic) Fluency scores, for example, are correlated with the degree of Aβ in multiple left frontal regions consistent with lesion and cerebral metabolism studies that have attributed verbal fluency to left frontal regions.^67–69^ However, Letter (phonemic) (letter) Fluency scores were not correlated with Aβ despite similar severity of impairment amongst the md-aMCI participants (Table 1) and despite evidence linking Letter Fluency to left frontal pathology.^67–69^ This may suggest that Letter Fluency deficits are more sensitive to other pathology than to Aβ deposition. This disparity may be reflective of the greater semantic (rather than phonemic) fluency deficits observed in early Alzheimer's disease.^70^

Another striking pattern that may reflect Aβ mediated local circuit dysfunction is the predominance of frontal associations of Aβ with short-term recall scores as compared with delayed recall scores (best represented in Fig. 2A). Episodic memory deficits are one of the earliest cognitive predictors of Alzheimer's disease.^71^ Immediate and delayed recall have been associated with distinct memory systems with immediate recall most impacted by frontal atrophy.^72^ Meanwhile, delayed recall in Alzheimer's disease is most strongly linked to hippocampal dysfunction.^71,72^ Whilst both measures seem to have similar value for predicting progression to Alzheimer's disease, immediate memory impairments occur earlier possibly reflecting the early deposition of Aβ in frontal regions.^73^ Greater correlation of immediate recall performance with Aβ in frontal regions is consistent with the role of frontal lobes in working memory and encoding.^72^

Finally, the observed correlation of visual-spatial immediate recall with Aβ in the visual association cortex may reflect Aβ mediated local circuit dysfunction. Multiple areas of the occipital and parietal cortex house redundant representations of visual working memory information, increasing fidelity of visual working memory when the primary visual cortex is distracted.^74–76^ Additionally, the frontal eye fields play a role in directing visual attention, and dysfunction caused by Aβ in this region may impair short-term visual-spatial recall.^77^ The supporting role of these regions in visual-spatial working memory may explain the restriction of Aβ correlations with short-term recall as opposed to delayed recall. Contrarily, previous studies of human and non-human primates have found the orbitofrontal cortex to be important in visual memory formation, particularly with tasks explicitly instructing memorization.^78–81^ Aβ mediated disruption of redundant or supportive encoding functions in the orbitofrontal cortex could underlie the correlation of Aβ in this region with delayed visual recall performance.

Aβ accumulation in regions associated with the default mode network (DMN) (i.e. the prefrontal cortex, angular gyrus, posterior cingulate cortex and precuneus) was correlated with neuropsychological performance on multiple measures. These observations might suggest that Aβ contributes (directly or indirectly) to early functional changes in the DMN affecting multiple domains of cognition to varying degrees. Structural MRI, diffusion tensor imaging (DTI) studies and fluorodeoxyglucose-PET studies find evidence that DMN disruption contributes to cognitive deficits in md-aMCI and Alzheimer's disease.^82,83^ Likewise, correlations of Aβ in the insula and dorsal cingulate regions with multiple cognitive measures may implicate Aβ-mediated dysfunction of the salience network in multiple cognitive deficits.

MCI is a heterogenous condition associated with a number of diseases and pathological processes.^84,85^ Even amongst those exhibiting evidence of preclinical Alzheimer's disease, it is difficult to say with certainty when or if an individual with MCI will progress to Alzheimer's disease.^85^ We chose to include all subjects in our analysis, regardless of Aβ status, as we thought this would be more representative of individuals with clinical MCI, some of whom are ‘Aβ negative’, as well as healthy elderly individuals, some of whom are ‘Aβ positive’. Likewise, the mechanisms linking Aβ and cognition or neurodegeneration could be direct or indirect (e.g. completely or partially mediated by tau or inflammatory processes). Applying the current Aβ and grey matter volume results to guide analysis of longitudinal follow-up in this cohort may provide better insight into the nature of the relationships between Aβ and neurodegeneration underlying the observed correlations. Longitudinal studies in both MCI and healthy older adults are critical to establish whether Aβ precedes or is followed by the neurodegeneration reflected by grey matter volume loss and to place these findings in the context of other ongoing pathological processes.

Previous studies have found md-aMCI to be associated with the highest rates of progression to Alzheimer's disease amongst all the MCI subtypes, with the proportion of high Aβ accumulators ranging from 77 to 83%.^27,30,31^ Moreover, higher proportion of people with md-aMCI (as opposed to sd-aMCI) exhibits other pathological markers observed in Alzheimer's disease, such as extensive grey matter atrophy and white matter disruptions.^30,82,86–90^ Using a previously validated mean cortical Aβ threshold of DVR > 1.2, we found that subjects with md-aMCI exceeded this threshold at twice the rate of comparison subjects (51.1 versus 21.6%). Meanwhile, we did not observe a statistically significant reduction in grey matter volume in md-aMCI subjects relative to healthy comparison subjects. Both observations may reflect the relatively young age of this sample; Aβ and grey matter volumes correlate with subject age (Fig. 1B and D). The lower estimates for the proportion of high Aβ accumulators in this sample may also be related to the use of a more stringent mean cortical Aβ threshold, the lower proportion of subjects with an *APOE4* allele and the larger sample size of our study relative to prior studies of Aβ in md-aMCI. Amongst healthy comparison subjects, the proportion of ‘Aβ positive’ individuals (at the threshold of DVR > 1.2) was consistent with prevalence estimates ranging from 20 to 40% based on other Aβ PET and post-mortem pathology studies.^8,38,91^

## Conclusion

Patterns of Aβ pathology and grey matter volume loss were correlated with performance on neuropsychological tests of immediate and delayed recall and Category, but not Letter Fluency. Future studies will evaluate how patterns of Aβ deposition correlate with cognitive decline longitudinally, in addition to characterizing how other aspects of Alzheimer's disease pathology (e.g. tau, synaptic markers) may influence these correlations.

## Supplementary Material

fcac016_Supplementary_Data

## Abbreviations

Aβ =: amyloid beta protein
BSR =: bootstrap ratio
BVMT-R =: Brief Visual Memory Test-Revised
CDR =: Clinical Dementia Rating
CVLT =: California Verbal Learning Test
DKEFS =: Delis–Kaplan Executive Function Scale
DVR =: distribution volume ratio
PLS =: partial least-squares
MCI =: mild cognitive impairment
md-aMCI =: multiple domain-amnestic mild cognitive impairment
MMSE =: Mini-Mental State Exam
MNI =: Montreal Neurologic Institute
