# Supplementary material for: Regional amyloid correlates of cognitive performance in ageing and mild cognitive impairment
Source: Brain Commun. 2022 Feb 7;4(1):fcac016. doi: 10.1093/braincomms/fcac016 (PMC8882008; doi:10.1093/braincomms/fcac016)
Supplement: fcac016_Supplementary_Data [file fcac016_supplementary_data.docx]

**Supplementary Table 1: Neuropsychological test performance by group**

|  | **Mild Cognitive Impairment Group**  **Mean ±SD** | **Healthy Comparison Group**  **Mean ±SD** | **F** | **p- value** |
| --- | --- | --- | --- | --- |
| WTAR Standard Score | 100.96 ± 15.76 | 105.68 ± 13.56 | 2.48 | 0.119 |
| DKEFS Trails Visual Scan Time | 26.28 ± 8.75 | 25.00 ± 8.94 | 0.45 | 0.503 |
| DKEFS Trails Number Sequencing Time | 45.28 ± 22.64 | 36.22 ± 11.50 | 5.15 | 0.026 |
| DKEFS Trails Letter Sequencing Time | 52.02 ± 33.17 | 36.17 ± 14.27 | 7.68 | 0.007 |
| DKEFS Trails Number and Letter Sequencing Time | 134.96 ± 88.85 | 93.72 ± 32.70 | 7.27 | 0.009 |
| DKEFS Fluency Letter Fluency | 37.47 ± 10.94 | 44.68 ± 12.51 | 7.97 | 0.006 |
| DKEFS Fluency Category Fluency | 33.83 ± 9.65 | 40.68 ± 8.24 | 12.40 | 0.001 |
| DKEFS Fluency Category Switching | 11.70 ± 3.20 | 13.86 ± 2.16 | 12.32 | 0.001 |
| DKEFS Fluency Total Switching Accuracy | 10.49 ± 3.33 | 12.92 ± 2.17 | 15.21 | 0.000 |
| DKEFS Free Sorting Confirmed Correct | 9.13 ± 2.41 | 10.05 ± 2.07 | 3.70 | 0.058 |
| DKEFS Free Sorting Description | 32.04 ± 10.37 | 36.41 ± 9.16 | 4.22 | 0.043 |
| DKEFS Sort Recognition Description | 28.47 ± 9.99 | 34.86 ± 7.98 | 10.35 | 0.002 |
| Symbol Digit Modalities Test: 30 seconds^a^ | 14.74 ± 3.74 | 16.84 ± 3.14 | 7.57 | 0.007 |
| Symbol Digit Modalities Test: 60 seconds^a^ | 26.45 ± 7.45 | 30.24 ± 5.72 | 6.80 | 0.011 |
| Symbol Digit Modalities Test: 90 seconds^a^ | 39.55 ± 10.64 | 45.73 ± 8.92 | 8.43 | 0.005 |
| Symbol Digit Modalities Test: Incidental Learning | 3.94 ± 2.40 | 5.41 ± 2.19 | 6.97 | 0.010 |
| CVLT Total Recall Trials 1-5 | 44.17 ± 12.20 | 55.22 ± 10.71 | 19.36 | 0.000 |
| CVLT Short Delay Free Recall | 7.89 ± 3.91 | 11.22 ± 3.17 | 17.96 | 0.000 |
| CVLT Short Delay Cued Recall | 9.47 ± 3.14 | 12.24 ± 3.02 | 16.29 | 0.000 |
| CVLT Long Delay Free Recall | 8.17 ± 3.92 | 11.89 ± 2.98 | 22.86 | 0.000 |
| CVLT Long Delay Cued Recall | 9.55 ± 3.24 | 12.46 ± 2.60 | 20.02 | 0.000 |
| WMS LMI Recall | 12.60 ± 3.35 | 15.81 ± 3.04 | 19.93 | 0.000 |
| WMS LMI Thematic Recall | 4.87 ± 1.23 | 5.59 ± 1.12 | 7.87 | 0.006 |
| WMS LMII Recall | 10.70 ± 4.23 | 14.95 ± 3.14 | 25.86 | 0.000 |
| WMS LMII Thematic Recall | 4.60 ± 1.56 | 5.68 ± 1.08 | 12.96 | 0.001 |
| BVMT-R Total Recall Trials 1-3 | 15.30 ± 7.84 | 18.92 ± 6.09 | 5.14 | 0.026 |
| BVMT-R Learning Trials 1-3 | 2.64 ± 1.94 | 4.19 ± 1.88 | 13.30 | 0.000 |
| BVMT-R Delayed Recall | 5.81 ± 3.17 | 7.68 ± 2.58 | 8.15 | 0.005 |

^a^Number correct

Abbreviations: BVMT-R, Brief Visual Memory Test-Revised; CVLT, California Verbal Learning Test; DKEFS, Delis-Kaplan Executive Function System; LM, Logical Memory; WTAR, Wechsler Test of Adult Reading; WMS, Wechsler Memory Scale.

**Supplementary** **Table 2: Cortical regions with greater^*^ Aβ in md-aMCI subjects relative to comparison group.**

|  | **Left Hemisphere** | | |  | **Right Hemisphere** | | | |  |
| --- | --- | --- | --- | --- | --- | --- | --- | --- | --- |
| **Region** | **MNI**  **Coordinates**  **X Y Z (mm)** | **Talairach**  **Coordinates X Y Z (mm)** | **Z-**  **Score** |  | | **MNI**  **Coordinates**  **X Y Z (mm)** | **Talairach**  **Coordinates**  **X Y Z (mm)** | **Z-**  **Score** | |
| **Frontal Cortex** |  |  |  |  | |  |  |  | |
| Orbital Operculum (BA 47) |  |  |  |  | | 44 30 -8 | 42 26 -3 | 3.82 | |
| Insula (BA 13) | -44 -10 0 | -42 -12 3 | 3.74 |  | | 38 20 -10 | 36 16 -4 | 3.75 | |
|  |  |  |  |  | |  |  |  | |
| **Temporal Cortex** |  |  |  |  | |  |  |  | |
| Sup. Temporal Gyrus (BA 21) | -64 -12 -4 | -60 -14 0 | 3.90 |  | |  |  |  | |
| Sup. Temporal Gyrus (BA 22) | -62 -14 0 | -58 -15 3 | 3.89 |  | | 64 -28 2 | 61 -28 6 | 3.78 | |
| Sup. Temporal Gyrus (BA 38) |  |  |  |  | | 52 12 -12 | 49 8 -5 | 4.10 | |
| Mid. Temporal Gyrus (BA 38) |  |  |  |  | | 50 8 -34 | 48 2 -25 | 3.76 | |
| Inf. Temporal Gyrus (BA 37) | -48 -60 -14 | -46 -61 -7 | 4.29 |  | |  |  |  | |
|  |  |  |  |  | |  |  |  | |
| **Parietal Cortex** |  |  |  |  | |  |  |  | |
| Precuneus (BA 7) | -48 -68 42 | -48 -65 40 | 3.82 |  | | 6 -68 50 | 6 -64 45 | 3.76 | |
| Posterior Cingulate (BA 31) | -8 50 36 | -7 -47 34 | 3.84 |  | | 8 -58 36 | 8 -55 34 | 4.06 | |
| Sup. Parietal Lobule (BA 7) | -18 -70 46 | -18 -66 42 | 4.33 |  | | 12 -64 56 | 12 -60 50 | 3.77 | |
| Sup. Parietal Lobule (BA 40) | -36 -38 50 | -36 -35 46 | 3.77 |  | |  |  |  | |
| Fusiform Gyrus (BA 37) | -36 -48 -24 | -34 -49 -16 | 4.32 |  | | 44 -56 -18 | 43 -57 -10 | 3.60 | |
| Inf. Parietal Lobule (BA 39) | -46 -62 34 | -46 -59 33 | 3.89 |  | | 46 -60 38 | 47 -57 36 | 3.77 | |
| Inf. Parietal Lobule (BA 40) | -42 -52 50 | -42 -49 46 | 3.81 |  | |  |  |  | |
|  |  |  |  |  | |  |  |  | |
| **Sub-Cortical Regions** |  |  |  |  | |  |  |  | |
| Putamen | -24 0 -6 | -23 -2 -1 | 4.04 |  | | 22 12 -4 | 21 9 0 | 3.81 | |

Abbreviations: BA, Brodmann area; MNI, Montreal Neurological Institute.

^*^Two sample *t*-tests in SPM12 evaluated between-group differences among md-aMCI and comparison subjects. The results are reported at a cluster-level, family-wise error (FWE) corrected threshold of *p* ≤ 0.001 and at a peak voxel uncorrected threshold of *p* ≤ 0.0001: Height threshold *p*=0.001 and extent threshold (k) =50 voxels. The cluster size (k_E_) is 50,461.
